# The Avantage ® dual mobility cup in primary total hip arthroplasty: A registry study

**DOI:** 10.1016/j.jor.2024.05.010

**Published:** 2024-05-16

**Authors:** E. Castiello, A. Bruschi, B. Bordini, F. De Gaetano, D. Tigani

**Affiliations:** a Department of Orthopaedic Surgery Ospedale Maggiore “Carlo Alberto Pizzardi”, Largo Nigrisoli 2, 40133, Bologna, Italy; b Department of Biomedical and Neuromotor Science-DIBINEM, IRCCS Rizzoli Orthopaedic Institute, University of Bologna, Via Zamboni 33, 40125, Bologna, Italy; c Medical Technology Laboratory, IRCCS – Rizzoli Orthopaedic Institute, Via di Barbiano 1/10, 40136, Bologna, Italy

**Keywords:** Dual mobility cup, Avantage cup, Total hip arthroplasty, Aseptic loosening, Dislocation, Intraprosthetic dislocation

## Abstract

**Background:**

Avantage Cup has been widely used in dual mobility implants. However, in Swedish Registry, the outcome of the Avantage Cup is reported with higher implants revision compared to control. The aim of our study was to verify if the same results are present in the Registry of Prosthetic Orthopedic Implants (RIPO) of Emilia Romagna (ER, Italy), as the Avantage cup was the most implanted dual mobility cup for a long follow-up reported in this Registry (2000–2012). Furthermore, we assessed the survival rate of the implant over the time.

**Methods:**

We included all patients that underwent a primary THA using the Avantage cup during the period 2000–2020 in RIPO Registry. The survivorship of the primary THA implants was calculated and plotted according to Kaplan-Meier method.

**Results:**

886 Avantage cups were included in the analysis. During the observational period 44 hips were revised. The most common reasons for revision were: periprosthetic fractures (PPF) (n = 7, 0.8 %), deep infection (n = 7, 0.8 %), and cup aseptic loosening (n = 13, 1.5 %).

The survival rate of the implant was 96.8 % (95.3–97.8) at 5 years, 95.7 at 10 years (94.0–97.0) and 92.1 at 15 years (88.5–94.6).

**Conclusion:**

In conclusion, this study has demonstrated that the Avantage cup in primary hip arthroplasty implanted with a “friendly” femoral stem granted satisfactory long-term survival. Therefore, in the Swedish Registry, the cause of the poor results presented for Avantage Cup could be the thick, rough neck stem of the widely used Lubinus stem.

## 1 Introduction

Dual mobility (DM) implants represent an appealing option for achieving hip stability, particularly in challenging primary and revision total hip arthroplasty (THA) cases. These implants have been utilized in Europe since the 1970s, primarily in scenarios involving a potentially or confirmed unstable hip. However, findings from registry-based studies regarding DM implants present conflicting results. While many reports suggest that using a dual mobility cup (DMC) as the primary treatment for hip fractures is linked to a reduced risk of revision, particularly due to dislocation, others indicate that THA with a dual mobility cup may yield outcomes comparable to conventional methods in terms of overall revisions, as well as revisions specifically attributable to dislocation or infections.^1^, ^2^, ^3^ Additionally, some studies indicate that THAs with dual mobility cups are more frequently revised due to infections.^3^, ^4^

According to data from various national registries, the Avantage cup (manufactured by Biomet, Warsaw, IN, USA) is among the most commonly utilized dual mobility cups.^5^^,^^6^ In the Swedish Hip Arthroplasty registry, the use of dual mobility cups was first reported in 2002 (primarily in revisions) and steadily increased until 2018, with around 500 revisions reported. However, this trend decreased thereafter, with approximately 370 insertions per year in 2020 and 2021, possibly reflecting a decline in performed revisions. Cemented DMCs have been the predominant choice in primary operations, but it has become increasingly common to cement a DMC into an existing acetabular cup during revisions or to convert a non-DMC to a DMC using a metal insert.

In 2018, unclear and concerning outcomes of the Avantage dual mobility cup were reported, indicating an elevated risk of revision.^3^ However, the reasons behind these outcomes remained unclear even in the latest report from 2022.^7^ Rogmark et al. suggested that the results from the Swedish Registry couldn't be solely explained by implant-related factors, as the Avantage cup was more frequently chosen for elderly and frail patients with femoral fractures.^3^

Our study aimed to determine if similar outcomes were observed in the Registry of Prosthetic Orthopedic Implants (RIPO) of Emilia Romagna (ER, Italy), as the Avantage cup was the most commonly implanted dual mobility cup with long-term follow-up data in this registry.^1^ Additionally, we assessed the implant's survival rate over time.

## 2 Methods

### 2.1 Study setting

The regional RIPO provided data on hip, knee and shoulder arthroplasties since January 2000 in the Italian region of Emilia-Romagna, Italian region counting about five million inhabitants. More than 60 orthopaedic departments are involved in the RIPO registry, with a completeness of 98 %. At 31st December 2020, data on nearly 145.000 total hip-replacements and 20.000 revisions have been collected in the Registry. More than 100 different types of commercially available hip prosthesis are registered to analyze their outcomes.

For the present study, we included all patients that underwent a primary THA using the Avantage cup during the period 2000–2020. The survivorship of the primary THA implants was calculated and plotted according to Kaplan-Meier method. The statistical analysis was performed using SPSS 14.0 for Windows, version 14.0.1 (SPSS, Chicago, Illinois, USA) and JMP, version 12.0.1 (SAS Institute, Cary, North Carolina, USA). Ethical approval for the study was not required as registry studies are covered by the informed consent signed at point of treatment.

### 2.2 Characteristics of the implant

The Avantage cup is a second-generation stainless steel DM cup with vacuum plasma spray titanium coating doped with hydroxyapatite (HA) on the surface. At the time of the first commercialization, the cup was just coated by HA. The Avantage cup is credited as the first press fit DM cup and subsequently as the primary DM specifically designed to be cemented, with a high polished surface and circular and radial grooves.

It has a cylindric-spherical design with flattened polar area. Its anatomic shape presents circumferential wins locate in the equatorial area to improve primary fixation on the inferior area and to limit impingement. This implant is on the market since 1998 with possibility of cemented and cementless fixation. Considering cementless fixation it is possible to choose between three different solutions: the Reload (standard press fit DM cup), the 3 P cup (characterized by the presence of ischial and pubic pegs combined with single flange for a Ø 4.5 mm cortical screw in the iliac wing) and the cementless revision cup (with an obturator hook, associated with superior plates for screws fixation options for additional stability).

### 2.3 Study population

Demographic data, primary diagnosis leading to THA, primary THA implant survival, perioperative complications, number and causes of failure, and data on revision procedures were collected. Revision was defined as the removal or change of any component. The implants were followed until the last date of observation (date of death or 31 December 2021). Closed reduction after a dislocation or incision and drainage for infection were not included in the RIPO. The median follow-up was 9.2 years (0–19.7).

## 3 Results

886 Avantage cups were included in the analysis. Among the primary implant cases 100 were cemented cups, whereas in 674 cases a press fit implant was used (Avantage reload); a tripod (Avantage 3P) solution was instead preferred in the remaining 112 hips (Table 1). In our Registry, the Avantage cup was the most used dual mobility implant during the period 2000–2012, whereas in the last report (2018), it has been overtaken by other implants Table 2 summarizes the types of constructs used as well as the year of implantation. Age at the time of operation ranged from 33 to 95 years (Table 3) and in total 61 % of patients were female and 39 % males (Table 4).

**Table 1:** Type of Avantage cup used.

Cup	N	%
**AVANTAGE RELOAD BIOMET**	391	44.1
**AVANTAGE BIOMET**	279	31.5
**AVANTAGE 3P BIOMET**	112	12.7
**AVANTAGE CEMENTED BIOMET**	101	11.4
**AVANTAGE REVISION BIOMET**	3	0.3
Total	**886**	**100.0**

**Table 2:**
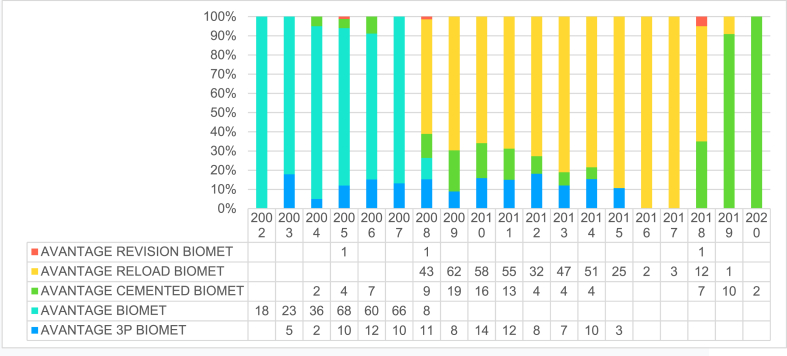
Year of operation and Type of Advantage cup.

**Table 3:** Distribution according to age.

Cup	Age (min-max)
**AVANTAGE 3P BIOMET**	72.9 (50–91)
**AVANTAGE BIOMET**	73.8 (51–95)
**AVANTAGE CEMENTED BIOMET**	77.2 (56–94)
**AVANTAGE RELOAD BIOMET**	71.4 (33–88)
**AVANTAGE REVISION BIOMET**	71.3 (57–82)
Total	**72.9 (33**–**95)**

**Table 4:** Gender distribution.

Cup	Sex			
	Female		Male	
	N	%	N	%
**AVANTAGE 3P BIOMET**	89	79.5	23	20.5
**AVANTAGE BIOMET**	173	62.0	106	38.0
**AVANTAGE CEMENTED BIOMET**	80	79.2	21	20.8
**AVANTAGE RELOAD BIOMET**	199	50.9	192	49.1
**AVANTAGE REVISION BIOMET**	2	66.7	1	33.3
Total**e**	**543**	**61.3**	**343**	**38.7**

During the observational period 44 hips were revised. The most common reasons for revision were: periprosthetic fractures (PPF) (n = 7, 0.8 %), deep infection (n = 7, 0.8 %), and cup aseptic loosening (n = 13, 1.5 %) (Table 5, Table 6).

**Table 5:** Implant failure analysis and follow up.

CUP	N operations	N failures	Follow-up (min-max)
**AVANTAGE 3P BIOMET**	112	7	9.8 (0–17.2)
**AVANTAGE BIOMET**	279	19	10.4 (0–19.7)
**AVANTAGE CEMENTED BIOMET**	101	1	7.5 (0.3–16.0)
**AVANTAGE RELOAD BIOMET**	391	17	8.5 (0–13.7)
**AVANTAGE REVISION BIOMET**	3	–	6.4 (2–13.2)
Total	**886**	**44**	**9.2 (0**–**19.7)**

**Table 6:** The table shows the rate of revision according to the cause of implant revision: the % distribution of the causes of failure is shown.

Primary implants			
Cause of implant revision	Rate	%	% Distribution of failure causes
Recurrent prosthesis dislocation	**2**/886	0.2	4.5
Periprosthetic bone fracture	**7**886	0.8	15.9
Septic loosening	**7**/886	0.8	15.9
Cup aseptic loosening	**13**/886	1.5	29.5
Total aseptic loosening	**1**/886	0.1	2.3
Stem aseptic loosening	**4**/886	0.5	9.1
Unknown (performed outside Emilia Romagna Region)	**6**/886	0.7	13.6
Pain without loosening	**3**/886	0.3	6.8
Unknown	**1**/886	0.1	2.3
**Total**	**44/**886	**5.0**	**100.0**

**Fig. 1:**
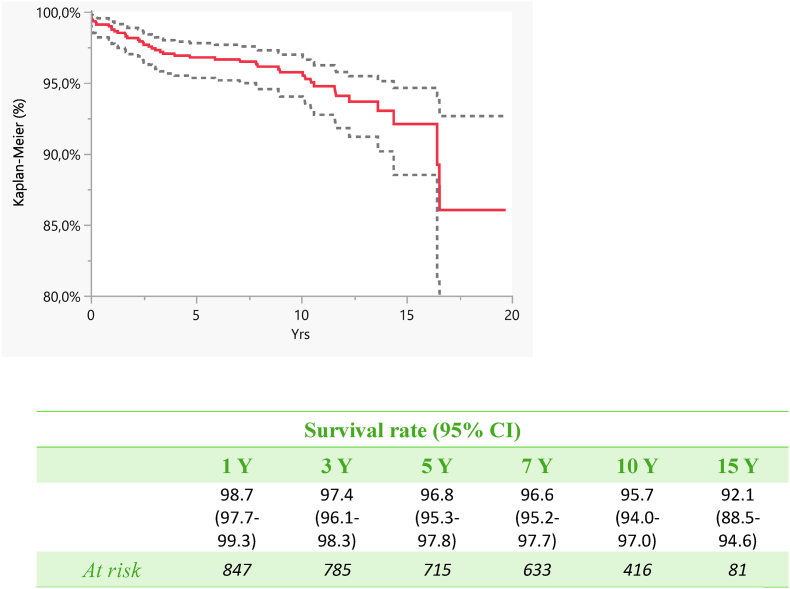
Survival analyses (Kaplan-Meier): Implants were followed until the last date of observation (date of death or 31st December 2020). The endpoint is the revision of the stem and/or the modular neck and/or cup.

**Fig. 2:**
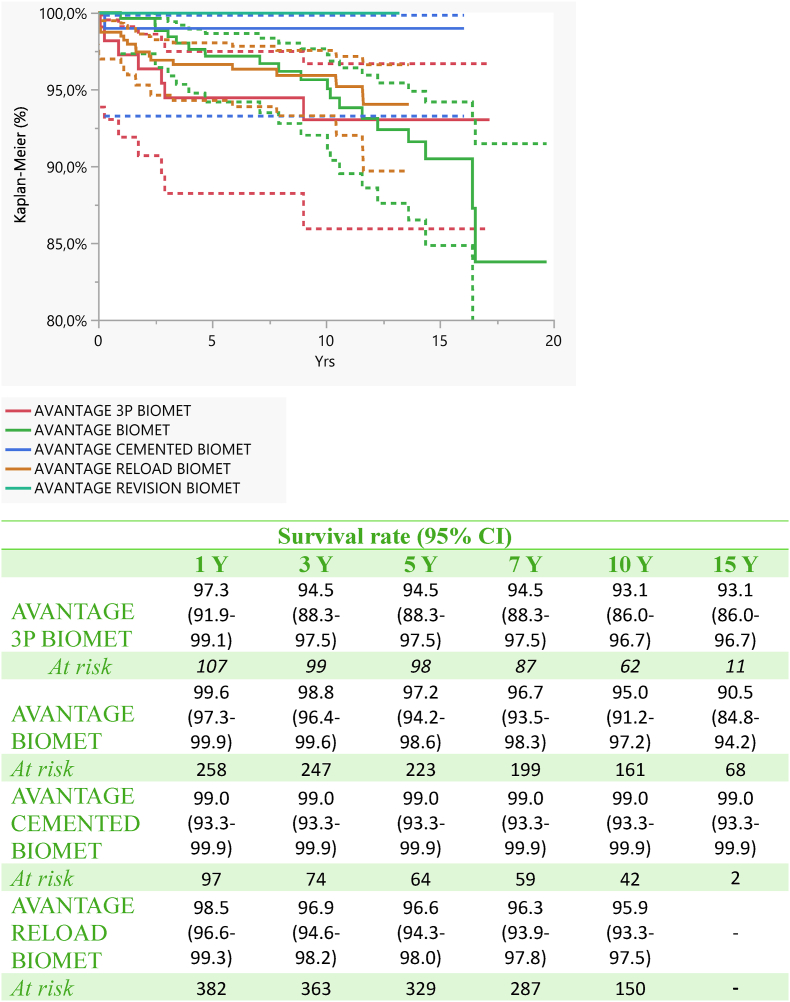
Survival analysis according to the different Advantage cups.

The survival rate of the implant was 96.8 % (95.3–97.8) at 5 years, 95.7 at 10 years (94.0–97.0) and 92.1 at 15 years (88.5–94.6) (Fig. 1, Fig. 2).

## 4 Discussion

To our knowledge, this is the first study on the Avantage dual mobility cup in primary total hip arthroplasty within a registry study. Our findings indicate that this implant choice exhibits favorable performance with a satisfactory long-term survival rate. The incidence of revision at 5, 10, and 15 years was found to be comparable to other alternatives.

While the Avantage cup enjoys widespread use in Europe,^1^^,^^2^^,^^5^ recent scrutiny from the 2018 Sweden National Registry report suggests that the cemented Avantage implant exhibits inferior outcomes compared to the control group.^2^ The exact reasons behind this disparity remain elusive, although the patient case mix likely contributes. Notably, this cup was more frequently selected for older patients with hip fractures in comparison to the control group. Interestingly, similar case mix patterns were observed with the other two dual mobility cups (ADES and Polar cup) but did not demonstrate inferior survival rates. However, it's worth mentioning that both ADES and the Polar cup were utilized in fewer cases and had shorter follow-up periods. Furthermore, the validity of the comparison may be compromised as certain comorbidities were not consistently recorded in the Swedish annual report of 2018.

Thus, our analysis focused on Avantage implant to present its outcomes within our Registry.

In our registry, the Avantage cup was frequently paired with members of the Taperloc stem family (including standard cementless stem, cemented, and microplasty), which are distinguished by their smooth and thin neck design. This combination was utilized in 722 patients, with a failure rate of 5.3 % (38 cases).

Among other stems used experiencing failures, we identified at least two brands with no “friendly” stem with the dual mobility cup: the Conus (Sulzer) with 1 failure (100 %) characterized by a grit-blasted surface and a large neck, and the S-Rom J(&J) with 1 failure out of 7 implants (14 %) featuring an unpolished neck with sharp angles.

On the contrary, a femoral stem is "friendly" for a dual mobility cup when it has a thin profile, high polish (roughness 0.1mμ) with a head-neck ratio of at least 2. Other desirable characteristics include a smooth surface devoid of corners and complete coverage of the taper; therefore, extra-long or skirted heads aren't recommended.

The significance of the femoral neck in implant survival became evident in 2003 through the research of Dr. Daniel Noyer. His study on mid-term outcomes of dual mobility cups (DMC) highlighted the influence of femoral stem neck design and surface characteristics.^8^ Dr. Noyer demonstrated that revisions for intra-prosthetic dislocation (IPD), occurring approximately 4 years post-implantation, were twice as likely with rough necks compared to polished ones. Recently, a biomechanical study highlighted the critical role of femoral neck characteristics in conjunction with dual mobility cups.^9^

IPD was frequently associated with first-generation designs but less so with "friendly neck" designs. Of particular interest is the paper by Aubriot et al., reporting a 0.7 % rate of IPD at 15 years.^10^^,^^11^ These findings potentially elucidate the subpar outcomes of the Avantage Cup reported in the Swedish Registry.^2^^,^^12^^,^^13^ Notably, the most utilized stem in the Swedish Registry, the Lubinus, features a rough and thick neck.

Despite the supportive data presented, this study has limitations. Primarily, it exclusively focuses on primary total hip arthroplasty (THA), whereas DMCs are more commonly employed in revision procedures and in high-risk patients during primary implantation. Additionally, functional outcomes and patient-reported outcome measures (PROMs) were not available in the RIPO Registry.

In conclusion, this study highlights that the Avantage cup, when coupled with a "friendly" femoral stem, exhibits satisfactory long-term survival in primary hip arthroplasty. If the poor outcomes reported in the Swedish registry with the Avantage cup are attributable to dislocation (true or intra-prosthetic), periprosthetic osteolysis, or loosening, these issues may be linked to wear of the polyethylene liner due to the rough and thick neck of the Lubinus stem. Further research is warranted to comprehensively understand the causes of Avantage DM cup failure, considering both PROMs and femoral stem characteristics.

## Funding information

The authors declare that no funds, grants, or other support were received during the preparation of this manuscript.

## Ethics approval

Ethical approval for the study was not necessary because the registry collects data as standard practice on all patients, using a format protecting their identity.

## Consent to participate

Consent to participate was not necessary because the registry collects data as standard practice on all patients, using a format protecting their identity.

## Consent to publish

Consent to publish was not necessary because the registry collects data as standard practice on all patients, using a format protecting their identity.

## Availability on data and materials

Data and materials of the present paper are part of the Emilia-Romagna regional register on orthopaedic arthroplasty implantation (RIPO)

## Declaration of competing interest

The Authors declare no competing interest to declare.

## References

[1] Tigani D., Castiello E., Moghnie A. (2023). Use of dual-mobility cup in primary total hip arthroplasties: an Italian regional register (RIPO) study on three thousand, seven hundred and ten cases. Int Orthop.

[2] Jobory A., Kärrholm J., Overgaard S. (2019). Reduced revision risk for dual-mobility cup in total hip replacement due to hip fracture: a matched-pair analysis of 9,040 cases from the nordic arthroplasty register association (NARA). J Bone Joint Surg.

[3] Rogmark C., Nauclér E. (2022). Dual mobility cups Do not reduce the revision risk for patients with acute femoral neck fracture: a matched cohort study from the Swedish arthroplasty register. Injury.

[4] Kreipke R., Rogmark C., Pedersen A.B. (2019). Dual mobility cups: effect on risk of revision of primary total hip arthroplasty due to osteoarthritis: a matched population-based study using the nordic arthroplasty register association database. J Bone Joint Surg.

[5] Bloemheuvel E.M., Van Steenbergen L.N., Swierstra B.A. (2019). Dual mobility cups in primary total hip arthroplasties: trend over time in use, patient characteristics, and mid-term revision in 3,038 cases in the Dutch arthroplasty register (2007–2016). Acta Orthop.

[6] Castiello E., Moghnie A., Tigani D., Affatato S. (2022). Dual mobility cup in hip arthroplasty: an in‐depth analysis of joint registries. Artif Organs.

[7] Qvistgaard M., Nåtman J., Lovebo J., Almerud-Österberg S., Rolfson O. (2022). Risk factors for reoperation due to periprosthetic joint infection after elective total hip arthroplasty: a study of 35,056 patients using linked data of the Swedish hip arthroplasty registry (SHAR) and Swedish perioperative registry (SPOR). BMC Muscoskel Disord.

[8] Noyer D., Caton J.H. (2017). Once upon a time.... Dual mobility: history. Int Orthop.

[9] Wegrzyn J., Longaray J., Baez R., Herrera L. (2022). Which femoral neck for a dual mobility cup? A biomechanical evaluation. Int Orthop.

[10] Lautridou C., Lebel B., Burdin G., Vielpeau C. (2008). Survie à 16,5 ans de recul moyen de la cupule, double mobilité, non scellée de Bousquet dans l’arthroplastie totale de hanche. Série historique de 437 hanches. Revue de Chirurgie Orthopédique et Réparatrice de l’Appareil Moteur.

[11] Aubriot J.H., Lesimple P., Leclercq S. (1993). [Study of Bousquet's non-cemented acetabular implant in 100 hybrid total hip prostheses (Charnley type cemented femoral component). Average 5-year follow-up]. Acta Orthop Belg.

[12] Castiello E., Amendola L., Barca P. (2020). Letter to the editor on “asymptomatic intraprosthetic dual mobility cup dislocation with increased metal ion levels.”. Arthroplasty Today.

[13] Guyen O., Pibarot V., Vaz G., Chevillotte C., Béjui-Hugues J. (2009). Use of a dual mobility socket to manage total hip arthroplasty instability. Clin Orthop Relat Res.

